# Influence of albumin and phase angle on the survival of patients with chronic kidney disease on hemodialysis: a prospective study

**DOI:** 10.1590/2175-8239-JBN-2024-0207en

**Published:** 2025-09-26

**Authors:** Alain León Sáez, Joane Severo Ribeiro, Camila Nery da Silva, Cristiane Bündchen, Elizete Keitel, Gilson Pires Dorneles, Catarina Bertaso Andreatta Gottschall, Alessandra Peres

**Affiliations:** 1 Universidade Federal de Ciências da Saúde de Porto Alegre, Porto Alegre, RS, Brazil.

**Keywords:** Albumin, Phase Angle, Oxidative Stress, Hemodialysis, Mortality Predictors

## Abstract

**Introduction:**

Hemodialysis (HD) prolongs the survival of patients with end-stage chronic kidney disease (CKD), but mortality remains high due to malnutrition and inflammation. This study aimed to investigate whether low albumin levels and a reduced phase angle (PA) predict higher mortality in HD patients.

**Methods:**

This prospective cohort study followed 82 HD patients for 36 months. Biological markers, including albumin and oxidative stress indicators, were measured before and after a single HD session upon study enrollment (time zero). PA was determined by bioimpedance analysis. Kaplan-Meier survival curves and Cox regression analyses were performed to assess the association between albumin, PA, and mortality.

**Results:**

Patients with PA <4 had a mean survival of 11.6 months, compared to 27.0 months for those with PA ≥ 4 (HR = 5.66; 95% CI: 2.44–13.12). Similarly, patients with albumin <3.9 g/dL had significantly lower survival rates at 6, 12, and 36 months compared to those with albumin ≥3.9 g/dL (HR = 4.39; 95% CI: 1.81–10.63).

**Conclusion:**

Low albumin levels and a reduced PA are strong predictors of mortality in HD patients. Routine monitoring and targeted interventions to improve these markers could enhance survival outcomes.

## Introduction

Chronic kidney disease (CKD) is a clinical syndrome secondary to a definitive alteration of the kidney’s function and structure^1^. Hemodialysis (HD) has improved the life expectancy of patients with end-stage CKD, but inflammation associated with malnutrition may occur^2^. Chronic inflammation in CKD is characterized by elevated levels of inflammatory markers such as C-reactive protein (CRP), interleukin-6 (IL-6), interleukin-1 (IL-1), and tumor necrosis factor-alpha (TNF-α), which contribute to oxidative stress and endothelial dysfunction^3^. The kidney is a highly metabolic organ, with abundant oxidation reactions in the mitochondria, making it vulnerable to oxidative stress damage. Several studies have shown that oxidative stress can accelerate the progression of CKD^4^. The generation of oxidative molecules, such as hydrogen peroxide (H_2_O_2_), by nitrogen oxides (NOXs) can trigger the activation of several other pro-oxidative enzymes, leading to a vicious cycle of redox dysfunction^4,5^.

Phase angle (PA) is calculated as the arctangent of the reactance to resistance ratio. It is independent of body height and weight and has been considered an indicator of membrane integrity and water distribution between intracellular and extracellular spaces^6^. Lower PA can be a potential measure of early diagnosis of malnutrition and has been considered a risk factor for mortality in HD individuals^7,8^.

This study aimed to investigate whether baseline albumin and PA levels and their acute variation after a single HD session predict mortality over a 36-month follow-up in patients with CKD.

## Methods

This is a cohort study with a 36-month follow-up conducted at the Dialysis Unit of the Hospital Santa Casa de Porto Alegre and the Universidade Federal de Ciências da Saúde de Porto Alegre (UFCSPA), Brazil. The study protocol was approved by the Ethics Committee of Santa Casa (Number 3.899.710).

This study included adult patients (≥18 years) of both genders who provided written informed consent to participate. As patients with metallic orthopedic prostheses, cardiac pacemakers, or amputated limbs cannot undergo bioimpedance analysis, they were excluded from the study. We also excluded patients with acute inflammatory disease and infection, patients taking anti-inflammatory medication, patients positive for human immunodeficiency virus (HIV), and nursing and pregnant women.

Samples were collected before and after a single hemodialysis session after the patient was included in the study (time zero). All participants were already undergoing dialysis treatment before enrollment, so they were prevalent hemodialysis patients. Patients were then followed for 36 months to assess the association between the biomarkers measured at time zero and survival. No additional samples were collected during the follow-up period, which represents a limitation of the study.

At the start of the follow-up, albumin, triglycerides, HDL, and C-reactive protein data were taken from the patient’s medical records. The cut-off value for albumin levels was 3.9 g/dL. Additionally, a secondary dataset including albumin levels measured at the end of the 36-month follow-up or closest to the time of death was used for a complementary survival analysis. Glucose was also collected at this point but was used solely to validate the diagnosis of diabetes. Furthermore, the variation (delta) in albumin and phase angle before and after the initial hemodialysis session (time zero) was calculated to explore immediate physiological responses and their association with survival.

**Figure 1:**
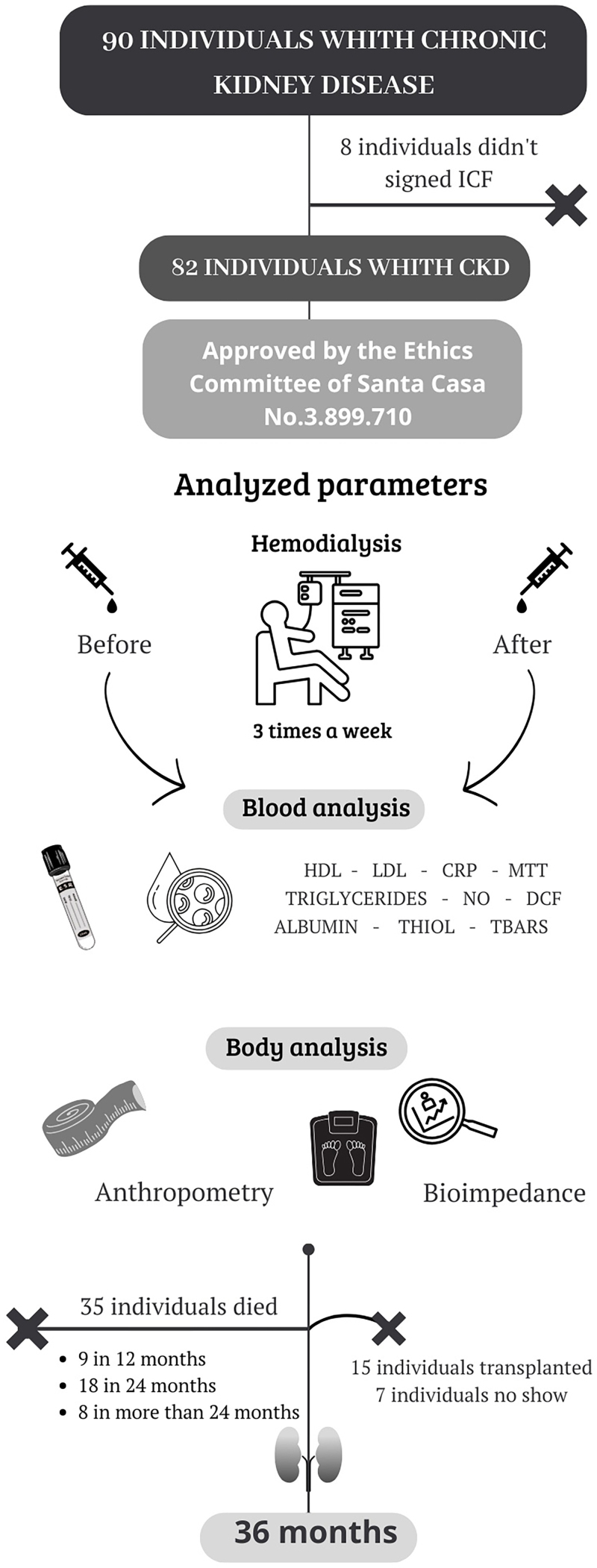
Design of the cohort study of patients with chronic kidney disease on hemodialysis. The study included 82 patients with chronic kidney disease who were followed for 36 months. Data were collected before and after the first hemodialysis session to evaluate biological markers and phase angle. Follow-up included assessment of albumin levels and patient survival. The primary outcome was mortality, and patients were followed until kidney transplantation, death, or study completion.

Bioimpedance analysis and blood were collected before and after the first hemodialysis session, right after study entrance. This material was used to analyze biological markers. After this collection (time zero), patients were followed up until 36 months for the outcome of death (Figure 1). A PA <4 was considered abnormal. Then, a questionnaire on the participant’s name, date of birth, phone number, gender, color/race, medical history of diseases, medication use, and alcohol intake was applied. In addition, self-reported previous infections were recorded.

For the bioimpedance analysis, the patient was informed about the pre-test procedure recommendations. The patient removed the shoe and sock on the side opposite the access and lay supine on a non-conductive surface with the head tilted back. The feet and upper inner thighs were positioned apart, as were the hands and the upper inner arms, ensuring no contact with the torso. Then, two electrodes were connected to the hand and two to the foot opposite the access site, after which the bioimpedance test was started. After the measurement, the data were recorded in the patient’s questionnaire.

Blood samples were collected by a qualified professional in the collection room of the dialysis unit using the hemodialysis access. After collection, the blood was centrifuged, and the plasma and erythrocyte samples were frozen at −20ºC for analysis of the oxidative profile.

Three hundred microliters of plasma was added to 600 μL of chilled (4°C) absolute ethanol to determine plasma nitrite levels. The mixture was vigorously shaken so that the ice-cold ethanol precipitated the proteins present in the plasma. After shaking, the plasma with ethanol was kept under refrigeration at 0°C for 30 minutes, and then the samples were centrifuged at 10,000 rpm for 5 minutes. Afterward, the supernatant was aliquoted and frozen for further analysis. The nitric oxide dosage was performed using Miranda’s method^9^, following the Griess assay. This method requires the assembly of two reagents (Reagent A: 0.5 g of naphthyl ethylenediamine dihydrochloride in 500 mL of distilled water and Reagent B: 5 g of sulfanilamide, 25 mL of phosphoric acid), which were mixed only on the day of the assay. A standard solution was created by adding 2 mg of sodium nitrite in 50 mL of distilled water (1.38 mL of the stock solution + 0.62 mL of water). Afterward, 100 μL of sample and working solution were pipetted into corresponding wells in a 96-well plate, and 100 μL of Griess’ reagent was added, incubated for 15 minutes, and read in a spectrophotometer at 550 nm.

Thiobarbituric acid reactive species (TBARS) levels were determined according to the method described by Ohkawa et al.^10^. Aliquots of serum and cell lysis (0.2 mL) were added to 250 µL of acetic acid (2.5M, pH 3.4) and 250 µL thiobarbituric acid (0.8%). This mixture was incubated for 90 minutes at 95°C and then centrifuged at 5,000 g for 15 minutes. The supernatant of the reaction was collected and analyzed in the microplate reader at 532 nm.

The plasma non-enzymatic antioxidant status was assessed through the reduction of the 3-[4,5-dimethylthiazol-2-y1]-2,5-diphenyltetrazolium bromide (MTT) by molecules with antioxidant action, according to Mosmann^11^. In a test tube, 100 µL of sample and 20 µL of MTT were added with 380 µL of phosphate-saline solution (10 mmol/L, pH 7.4). This solution was incubated in a water bath for 60 min at 37°C, protected from light. Then, 1 mL of an isopropyl alcohol solution with hydrochloric acid (0.4 N) was added and centrifuged at 3500 rpm for 10 min at room temperature. The reaction supernatant was transferred to a 96-well plate and analyzed in the microplate reader (Spectra Max 250; Molecular Devices) at 570 nm. The evaluation of the antioxidant status was expressed according to the absorbance obtained.

The ROS content was evaluated in plasma through the fluorescence intensity of the redox‐sensitive dye 2′,7′‐dichlorodihydrofluorescein diacetate (DCFH, 100 µM; Sigma‐Aldrich). DCFH can cross the cell membrane, and it undergoes the process of deacetylation by intracellular esterase enzymes, forming the intermediate compound 2′,7′‐dichlorodihydrofluorescein in the cytoplasm, which reacts with ROS forming oxidized 2′,7′-dichlorofluorescein (DCF), a fluorescent molecule. The assay involved adding 30 µL of sample, 30 µL of phosphate-saline solution (10 mmol/L, pH 7.4), and 24 µL of DCF (10 µM) into a 96-well plate. The plate was incubated for 30 minutes at 37°C. Fluorescence was measured using a microplate reader (Spectra Max 250; Molecular Devices; USA) with excitation and emission wavelengths set at 485 and 535 nm, respectively. The amount of intracellular ROS was expressed as fluorescence intensity.

### Statistical Analysis

Normality was checked using the Kolmogorov-Smirnov test. Qualitative variables were described by absolute and relative frequency, and quantitative variables by mean ± standard deviation and median.

The Wilcoxon test was used to compare before and after hemodialysis markers, and the McNemmar Test was used to compare before and after PA. To establish the albumin cut-off point that predicted death, the ROC curve was used. For patient survival analysis, the follow-up time was calculated in months from the date of BIA/evaluation to the date of death/transplantation/closure. The outcome of interest was death, and the censuses were transplanted and alive. Patients who dropped out, were transferred, or were missing for other reasons were not included in this analysis, as there was no record of the date. Kaplan-Meier curves were generated using the log-rank test to compare survival according to the factors of interest. Hazard ratio estimates with 95% CI were obtained using Cox regression analysis. For the multivariate analysis, variables with p-values <0.10 were selected from the univariate analysis and had limited missing data. Additional Cox regression models were developed using the final or last recorded values of albumin and glucose to explore late-stage biochemical predictors of mortality. Patients were categorized based on standard clinical cut-off points: albumin <3.9 g/dL and glucose ≥126 mg/dL. Furthermore, the delta (post – pre) albumin and PA were compared between survivors and non-survivors using descriptive and graphical analysis, including violin plots and grouped bar charts. Statistical analyses were conducted using the SPSS 25.0 statistical program (SPSS Inc., USA). The significance level adopted was 0.05.

## Results

The present study analyzed 82 patients (51.2% male) with CKD undergoing hemodialysis. After 36 months of follow-up, 15 stopped treatment due to kidney transplantation, 7 were transferred, and 35 died. The associated comorbidities were hypertension (91.5%), heart failure (17.1%), and obesity (24.4%). The average survival time in patients with diabetes was 20.3 months, and without diabetes was 26.4 months; patients with diabetes had a 22.6% higher mortality rate than the group without diabetes. Among the 42 patients with glucose values available at the end of follow-up, 11 had a prior diagnosis of diabetes mellitus. These individuals had a mean final glucose of 151.4 ± 49.9 mg/dL, while non-diabetics (n = 31) presented a mean of 95.9 ± 46.2 mg/dL. These findings support the consistency between the clinical diagnosis and biochemical markers of glycemic status.

The survival rate of non-diabetic and diabetic patients was 94.7 and 78.6% at six months, 81 and 64.3% at 12 months, 58.3 and 35.7% at 24 months, and 49.7 and 28.6% at 36 months. Table 1 describes the general characterization of the sample collected at time zero, i.e., pre-dialysis at the beginning of the follow-up.

**Table 1:** Baseline characteristics of the patients with CKD

Variables	n	Mean ± SD	Median (minimum–maximum)
Age (years)	82	52.5 ± 17.4	54.5 (20–84)
Dialysis time (months)	82	54.2 ± 51.6	40.5 (3–202)
After dialysis weight (Kg)	82	68.4 ± 19.7	63.1 (36.8–119.8)
After dialysis BMI (Kg/m^2^)	82	25.9 ± 5.9	25 (16.6–41.9)
Albumin (g/dL)	82	3.8 ± 0.3	3.8 (2.9–4.6)
C-reactive protein (mg/dL)	65	12.2 ± 15.3	7.8 (0.23–84)
PCR/albumin	65	3.2 ± 4.0	1.8 (0.1–22.1)
Triglycerides (mg/dL)	79	148.2 ± 88.5	125 (15–515)
HDL (md/dL)	79	42.1 ± 15.6	39 (11–88)
TRIG/HDL	79	4.2 ± 3.4	3.3 (0.4–21.7)

Abbreviations – SD: Standard Deviation; kg: kilogram; BMI: Body Mass Index; PCR: Reactive C Protein; HDL: High Density Lipoprotein.

Table 2 shows the variations found in the biological markers analyzed before and after the hemodialysis session at time zero. The thiol marker showed a significant increase (p = 0.001), the MTT marker had a significant reduction (p = 0.031), and the TBARS marker also showed a significant decrease (p < 0.001). In contrast, the DCF marker showed a significant increase (p = 0.046). The nitric oxide marker showed no significant difference. Table 2 also shows a significant increase in PA in patients after hemodialysis.

**Table 2:** Comparison of oxidative damage markers and phase angle before and after the hemodialysis session at baseline

Marker	N	Before	After	p
		Median (Min–Max)	Median (Min–Max)	
Thiols (nmol)	73	14.9 (3.3; 45.8)	17.8 (4.9; 61.1)	**0.001**
MTT (O.D.)	75	0.39 (0.15; 1.92)	0.32 (0.12; 2.18)	**0.031**
TBARS (nmol/mL)	73	552.4 (12.4; 1377.7)	214.1 (15.3; 1108.2)	**<0.001**
DCF (fluorescence intensity)	73	295.8 (195; 568.2)	321.8 (116.8; 888.3)	**0.046**
NO (mg/L)	39	40.3 (29.3; 65)	39.7 (27.9; 73.1)	0.132
Phase angle	82	5.2 (2.4; 7.7)	5.9 (2.7; 10.6)	**<0.001**

Abbreviations – DCF: Dichlorodihydrofluorescein; TBARS: Thiobarbituric acid reactive substances; MTT: (3-(4,5-dimethylthiazol-2-yl)-2,5-diphenyltetrazolium bromide); NO: Nitric oxide. Notes – p: Wilcoxon test; Significance level = 0.05.

Furthermore, 11.0% of patients had an altered PA (<4) at the beginning of the follow-up. Before hemodialysis, 14 patients (17.1%) had a PA < 4, and after the hemodialysis session, 9 (11%) remained with a PA < 4 (p = 0.063). However, more than 1/3 (5/14) of the patients who had a PA < 4 improved. The average survival time of patients with PA < 4 was 11.6 months (95%CI: 6.5–16.8), and that of patients with PA ≥ 4 was 27.0 months (95%CI: 24.2–29.8), p < 0.001, with a significant difference (log-rank = 11.5; p < 0.001; HR =5 .66 (95%CI: 2.44–13.12). Survival rates in PA < 4 and PA ≥ 4 were 66.75% × 95.1%, 55.6% × 80.9%, 11.1% × 60.2%, and 11.1% × 50.6% at 6, 12, 24, and 36 months, respectively. Table 3 shows the variables that significantly contributed to the hazard ratio of death over the 36 months.

**Table 3:** Biomarkers that contribute significantly to the hazard ratio considering the phase angle before and after hemodialysis

		Univariate				Multivariate			
		p	HR	95%CI		p	HR	95%CI	
phase angle before hemodialysis	Age (each 5 years)	0.003	1.19	1.06	1.35	0.172	1.10	0.96	1.27
	Diabetes	0.091	1.88	0.90	3.93	0.692	0.84	0.34	2.03
	Post PA < 4	**0.000**	**5.99**	**2.80**	**12.79**	**0.042**	**2.69**	**1.03**	**7.01**
	DCF pos (each 50 uM)	0.076	0.87	0.75	1.01	0.220	0.90	0.76	1.07
	Albumin < 3.9	**0.001**	**4.39**	**1.81**	**10.63**	**0.017**	**3.40**	**1.24**	**9.30**
phase angle after hemodialysis	Age (each 5 years)	0.003	1.19	1.06	1.35	0.122	1.12	0.97	1.31
	Diabetes	0.091	1.88	0.90	3.93	0.638	0.81	0.33	1.96
	Pre PA < 4	**0.000**	**5.66**	**2.44**	**13.12**	0.285	1.92	0.58	6.37
	DCF pos (each 50 uM)	0.076	0.87	0.75	1.01	0.174	0.89	0.75	1.05
	Albumin < 3.9	**0.001**	**4.39**	**1.81**	**10.63**	**0.008**	**3.81**	**1.41**	**10.25**

Abbreviations – DCF: Dichlorodihydrofluorescein; PA: phase angle; HR: hazard ratio. Notes – pre: before the hemodialysis; post: after the hemodialysis; Albumin < 3.9: cut-off; p: univariate or multivariate analysis; CI95%: confidence interval 95%; Significance level = 0.05.

The average survival time in patients with albumin <3.9 was 21.2 months (95%CI 17.8–24.7), and that of patients with albumin ≥3.9 was 31.7 months (95%CI 28.2–35.2), with a significant difference (log-rank = 13.0; p < 0.001; HR = 4.39; 95%CI: 1.81–10.63). Furthermore, 57.3% had albumin <3.9 (AUC = 0.742, 95%CI 0.635–0.849, p < 0.001, sensitivity = 82.7%, specificity = 61.7%). As for survival, the rates at 6, 12, 24, and 36 months for patients with albumin ≥3.9 and <3.9 were 96.6 and 88.2%, 88.5 and 70.7%, 84.1 and 35.4%, and 75.0 and 27.6%, respectively.

In a secondary analysis using values measured closest to death or at the end of follow-up, patients with albumin levels <3.9 g/dL had a significantly increased mortality risk (HR = 2.66; p < 0.001). These findings reinforce the prognostic value of albumin at baseline and in the late stages of follow-up and confirm the classification of diabetes using terminal glycemic values. While albumin showed minor fluctuations throughout the follow-up, its terminal values were similar to baseline (mean: 3.78 ± 0.73 g/dL, median: 3.90, range: 1.3–5.4 g/dL). Although glucose was not included in the survival models, its final values supported the diagnosis of diabetes mellitus in patients classified as diabetic.

**Figure 2:**
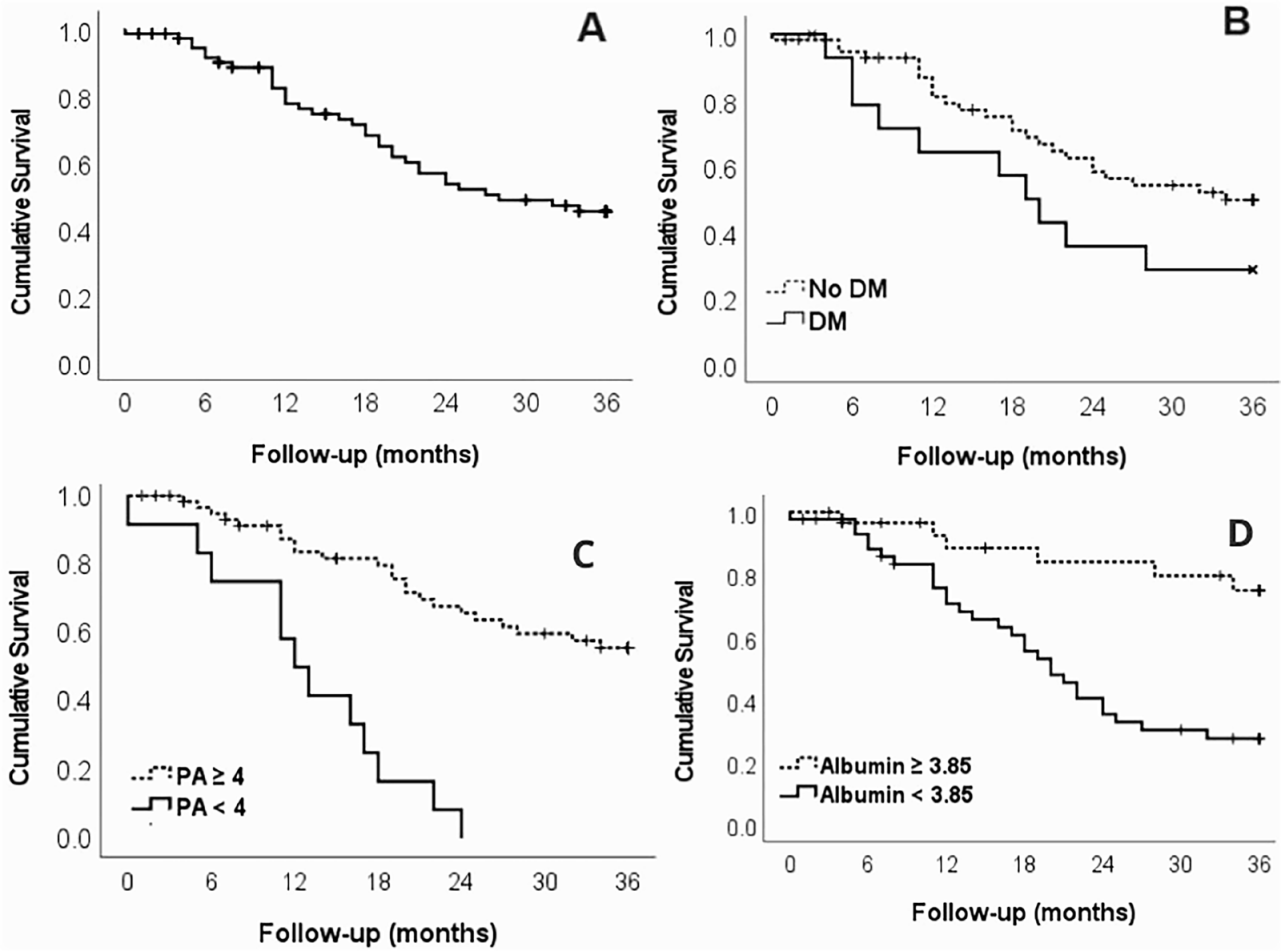
Kaplan-Meier survival curves at 36 months for patients with chronic kidney disease on hemodialysis. (A) Overall survival rate of the cohort; (B) Survival comparison between diabetic (DM) and non-diabetic patients; (C) Survival stratified by phase angle (PA) cut-off point, showing significant differences between patients with PA < 4 and PA ≥ 4. Statistical significance was set at p < 0.05 for all comparisons.

There was no significant difference in survival curves according to DM (log-rank = 3.0; p = 0.083; HR = 1.88; 95% CI: 0.90–3.93). The average survival time in patients with DM was 20.3 months (95% CI:14.1–26.6), and that of patients without DM was 26.4 months (95% CI: 23.4–29.5). These findings highlight the prognostic relevance of terminal hyperglycemia, even in patients not previously classified as diabetic. Figure 2 shows the overall survival over 36 months.

Among the 35 patients who died, 30 had a known cause of death, which was categorized by etiology: cardiovascular/circulatory collapse (n = 11), infectious/inflammatory (n = 10), neoplastic (n = 3), renal/access failure (n = 2), gastrointestinal/hemorrhagic (n = 1), end-of-life/palliative (n = 1), and unclassified (n = 3). The mean time to death varied by cause, with shorter survival in infectious and cardiovascular deaths (16.5 and 17.7 months, respectively) and longer times in neoplastic or hemorrhagic events.

## Discussion

During patient follow-up, the survival rate was 91.4% at 6 months, 77.6% at 12 months, 53.5% at 24 months, and 45.2% at 36 months. These survival rates were similar to those described by the Brazilian Dialysis Census where the survival rate described for the year 2021 was 77.7%^12^.

A significant difference was found in the parameters thiols, TBARS, and MTT when comparing between the pre-dialysis with post-dialysis measurements, as shown in Table 2. Impaired homeostasis of blood thiols has been commonly described in chronic kidney disease, with the highest expression level in end-stage patients on hemodialysis therapy. The factors involved in abnormal thiol levels may include impaired thiol metabolism in the kidney and other tissues exposed to uremic toxins, as well as metabolic consequences of uremic comorbidity, including chronic inflammation, malnutrition, anemia, and oxidative stress^13^.

TBARS determination is widely used for monitoring lipid peroxidation. Our study found a significant reduction in TBARS levels when comparing pre-and post-dialysis moments. Hemodialysis had an important effect in reducing oxidative stress in our work, unlike the study by Zargari and Sedighi^14^ in which the highest TBARS concentrations were found after HD. Another study, carried out by Schettler et al., also found an increase in TBARS levels after hemodialysis. However, when TBARS concentrations were corrected for plasma cholesterol to account for hemoconcentration, there was a significant decrease in TBARS due to dialysis treatment both in venous and arterial plasma^15,16^. Our findings revealed an increase in DCF levels post-hemodialysis, which may be attributed to uremia-induced eryptosis, a phenomenon characterized by erythrocyte shrinkage, membrane blebbing, and transfer of phosphatidylserine to the outer membrane leaflet^17,18^. After the hemodialysis session, the MTT assay indicated that oxidant levels were significantly decreased, which could be related to a decrease in oxidation levels since MTT can detect superoxide generation^19^.

The survival rate of patients with a PA lower than 4° - a marker of malnutrition for all patients regardless of sex - was also evaluated. Survival in this group was 66.7% at 6 months, 55.6% at 12 months, and 11.1% at 24 months. In the group that did not present malnutrition, survival was 95.1%, 80.9% and 60.2%, respectively. The parameter that showed the highest association with mortality was malnutrition, measured by PA (p < 0.001). In a study by Ruperto and Barril^20^, a PA lower than 4° was found to be predictor of mortality, with more than 70% of non-survivors having PA < 4°^21,22^. Zeni et al.^21^, in a study carried out in the same center and with the same patients, found that the PA remained associated with death after adjusting for age, extracellular water-to-total body water ratio, race, heart failure, obesity, diabetes, hypertension, and HDL cholesterol^21^.

Because the PA evaluates the electrical function of cell membranes, a low PA is associated with cell death or reduced cell function and is also a sign of sarcopenia and malnutrition, which can significantly impact the quality of life and survival of patients on dialysis^23,24,25,26^. These findings suggest that the PA may be one of the best predictors of mortality in hemodialysis patients. If this deficiency were corrected, mortality could be reduced by 50% over 24 months.

The survival of patients with diabetes in the USRDS in 2011 was 92.1% at three months, 78.3% at 12 months, and 66.8% at 24 months^27^. In the work by Silva et al., it was shown that survival for non-diabetics was different than for diabetics: 92% versus 87% in 1 year, 87% versus 77% in 2 years, and 69% versus 50% in 5 years^28^. Zeng et al.^26^ conducted a study with 87 hemodialysis patients, 33 of whom were diabetic and 54 were not. After one year of hemodialysis, there was an increase in hospitalizations due to heart failure, but without significant differences in mortality^26^.

Albumin was the only independent variable that significantly influenced patient mortality. Low serum albumin in peritoneal dialysis decreases survival in end-stage kidney disease patients^29^, indicating that high serum albumin level variation is associated with increased all-cause mortality^30^. In the present study, the risk of death during follow-up was increased by 4.4 and 3.8 times when albumin was lower than 3.9 g/dL in the univariate and multivariate analyses, respectively. In the Fulks et al.^31^ study, albumin levels >4.8 g/dL were associated with 15–18% relative risk reductions for mortality in an unselected population of men aged 20–49. The Outcomes and Practice Patterns Study (DOPPS)^32,33,34^ also suggests that >60% of HD patients have albumin levels <4.0 g/dL. Low serum concentrations of albumin have consistently been shown to be a powerful predictor of morbidity and mortality in CKD populations. Overall, maintaining adequate serum albumin levels and minimizing fluctuations could play a crucial role in improving the survival outcomes of hemodialysis patients.

We also explored the impact of late-stage biochemical markers on mortality. Low albumin and hyperglycemia close to death remained strongly associated with reduced survival, reinforcing the importance of routine metabolic monitoring throughout follow-up.

Additionally, we observed that patients with better acute responses to the initial hemodialysis session were more likely to survive, as measured by increased PA and mild rises in albumin. These findings suggest that long-term metabolic control and immediate physiological resilience play a role in survival outcomes in HD patients.

Regarding the cause of death, the most common etiologies were cardiovascular and infectious, consistent with the existing literature, and associated with earlier mortality. The absence of structured reporting in medical records limited a more detailed stratification, highlighting the need for better clinical documentation in future studies.

## Data Availability

The datasets generated and analyzed during the current study are not publicly available due to privacy and ethical restrictions involving patient data but are available from the corresponding author upon reasonable request and with appropriate institutional approvals.
