# Supplementary material for: Influence of albumin and phase angle on the survival of patients with chronic kidney disease on hemodialysis: a prospective study
Source: J Bras Nefrol. 2025 Sep 26;47(4):e20240207. doi: 10.1590/2175-8239-JBN-2024-0207en (PMC12477825; doi:10.1590/2175-8239-JBN-2024-0207en)
Supplement: Translation

# Translation: Influência da albumina e do ângulo de fase na sobrevida de pacientes com doença renal crônica em hemodiálise: um estudo prospectivo

**DOI:** 10.1590/2175-8239-JBN-2024-0207pt

**Authors:** Alain León Sáez, Joane Severo Ribeiro, Camila Nery da Silva, Cristiane Bündchen, Elizete Keitel, Gilson Pires Dorneles, Catarina Bertaso Andreatta Gottschall, Alessandra Peres

**Affiliations:** 1 Universidade Federal de Ciências da Saúde de Porto Alegre, Porto Alegre, RS, Brasil.

**Keywords:** Albumina, Ângulo de Fase, Estresse Oxidativo, Hemodiálise, Preditores de Mortalidade

## Abstract

**Introdução:**

A hemodiálise (HD) prolonga a sobrevida de pacientes com doença renal crônica (DRC) em estágio V, mas a mortalidade permanece elevada devido à desnutrição e à inflamação. Este estudo teve como objetivo investigar se baixos níveis de albumina e um ângulo de fase (AF) reduzido predizem maior mortalidade em pacientes em HD.

**Métodos:**

Este estudo de coorte prospectivo acompanhou 82 pacientes em HD por 36 meses. Marcadores biológicos, incluindo albumina e indicadores de estresse oxidativo, foram mensurados antes e após uma única sessão de HD no momento da inclusão no estudo (tempo zero). O AF foi determinado por análise de bioimpedância (BIA). Curvas de sobrevida de Kaplan-Meier e análises de regressão de Cox foram realizadas para avaliar a associação entre albumina, AF e mortalidade.

**Resultados:**

Pacientes com AF < 4 apresentaram sobrevida média de 11,6 meses, em comparação a 27,0 meses entre aqueles com AF ≥ 4 (HR = 5,66; IC 95%: 2,44–13,12). De forma semelhante, pacientes com albumina < 3,9 g/dL apresentaram taxas de sobrevida significativamente mais baixas aos 6, 12 e 36 meses em comparação àqueles com albumina ≥ 3,9 g/dL (HR = 4,39; IC 95%: 1,81–10,63).

**Conclusão:**

Baixos níveis de albumina e AF reduzido são fortes preditores de mortalidade em pacientes em HD. O monitoramento de rotina e intervenções direcionadas para melhorar esses marcadores podem contribuir para melhores desfechos de sobrevida.

## Introdução

A doença renal crônica (DRC) é uma síndrome clínica secundária a uma alteração definitiva da função e da estrutura dos rins^1^. A hemodiálise (HD) elevou a expectativa de vida dos pacientes com DRC em estágio V; no entanto, pode haver inflamação associada à desnutrição^2^. A inflamação crônica na DRC é caracterizada por níveis elevados de marcadores inflamatórios, como proteína C reativa (PCR), interleucina-6 (IL-6), interleucina-1 (IL-1) e fator de necrose tumoral alfa (TNF-α), os quais contribuem para o estresse oxidativo e a disfunção endotelial^3^. O rim é um órgão altamente metabólico, com abundantes reações de oxidação nas mitocôndrias, tornando-o vulnerável a danos causados pelo estresse oxidativo. Diversos estudos demonstraram que o estresse oxidativo pode acelerar a progressão da DRC^4^. A geração de moléculas oxidantes, como o peróxido de hidrogênio (H_2_O_2_), a partir de óxidos de nitrogênio (NOXs), pode desencadear a ativação de várias outras enzimas pró-oxidantes, levando a um ciclo vicioso de disfunção redox^4,5^.

O ângulo de fase (AF) é calculado como o arco tangente da relação entre reatância e resistência. É independente da altura e do peso corporal e tem sido considerado um indicador da integridade da membrana e da distribuição de água entre os compartimentos intra e extracelular^6^. Um AF menor pode constituir uma medida potencial para o diagnóstico precoce da desnutrição e tem sido considerado um fator de risco para a mortalidade em indivíduos em HD^7,8^.

Este estudo teve como objetivo investigar se os níveis basais de albumina e AF e sua variação aguda após uma única sessão de HD predizem a mortalidade ao longo de um acompanhamento de 36 meses em pacientes com DRC.

## Métodos

Trata-se de um estudo de coorte com acompanhamento de 36 meses realizado na Unidade de Diálise do Hospital Santa Casa de Porto Alegre e na Universidade Federal de Ciências da Saúde de Porto Alegre (UFCSPA), Brasil. O protocolo do estudo foi aprovado pelo Comitê de Ética da Santa Casa (número 3.899.710).

Este estudo incluiu pacientes adultos (≥18 anos), de ambos os sexos, que forneceram consentimento informado por escrito para participar. Como pacientes com próteses ortopédicas metálicas, marcapassos cardíacos ou membros amputados não podem ser submetidos à análise de bioimpedância, estes foram excluídos do estudo. Também excluímos pacientes com doença inflamatória aguda e infecção, pacientes em uso de medicação anti-inflamatória, pacientes positivos para o vírus da imunodeficiência humana (HIV) e mulheres grávidas e lactantes.

As amostras foram coletadas antes e depois de uma única sessão de hemodiálise, posteriormente à inclusão do paciente no estudo (tempo zero). Todos os participantes já estavam em tratamento dialítico antes da inclusão, sendo, portanto, pacientes prevalentes em hemodiálise. Os pacientes foram acompanhados por 36 meses para avaliar a associação entre os biomarcadores mensurados no tempo zero e a sobrevida. Nenhuma amostra adicional foi coletada durante o período de acompanhamento, o que representa uma limitação do estudo.

No início do acompanhamento, os dados relativos à albumina, triglicerídeos, HDL e proteína C reativa foram obtidos dos prontuários médicos dos pacientes. O valor de corte para os níveis de albumina foi de 3,9 g/dL. Além disso, um conjunto de dados secundário, incluindo os níveis de albumina medidos no final do acompanhamento de 36 meses ou mais próximo ao momento dos óbitos, foi utilizado para uma análise complementar de sobrevida. A glicose também foi coletada neste momento, mas foi utilizada exclusivamente para validar o diagnóstico de diabetes. Além disso, calculou-se a variação (delta) na albumina e no ângulo de fase antes e após a sessão inicial de hemodiálise (tempo zero) para explorar as respostas fisiológicas imediatas e sua associação com a sobrevida.

A análise da bioimpedância e a coleta de sangue foram realizadas antes e após a primeira sessão de hemodiálise, logo após a inclusão no estudo. Esse material foi utilizado para analisar marcadores biológicos. Após essa coleta (tempo zero), os pacientes foram acompanhados por até 36 meses para o desfecho de óbito (Figura 1). Um AF < 4 foi considerado anormal. Em seguida, foi aplicado um questionário para obter informações sobre o nome, data de nascimento, número de telefone, sexo, cor/raça, histórico médico de doenças, uso de medicamentos e consumo de álcool dos participantes. Além disso, foram registradas infecções prévias autorreferidas.

**Figura 1:** Desenho do estudo de coorte de pacientes com doença renal crônica em hemodiálise. O estudo incluiu 82 pacientes com doença renal crônica, acompanhados por 36 meses. Os dados foram coletados antes e após a primeira sessão de hemodiálise para avaliar os marcadores biológicos e o ângulo de fase. O acompanhamento incluiu avaliação dos níveis de albumina e sobrevida dos pacientes. O desfecho primário foi a mortalidade, e os pacientes foram acompanhados até o transplante renal, o óbito ou a conclusão do estudo.

Para a análise de bioimpedância, o indivíduo foi informado sobre as recomendações do procedimento pré-teste. O paciente tirou o calçado e a meia do lado oposto ao acesso e posicionou-se em decúbito dorsal sobre uma superfície não condutora, com a cabeça reclinada para trás. Os pés e a parte interna superior das coxas foram posicionados afastados, assim como as mãos e a parte interna superior dos braços, garantindo que não houvesse contato com o tronco. Em seguida, dois eletrodos foram conectados à mão, e dois ao pé oposto ao local do acesso, sendo iniciado o teste de bioimpedância. Após a medição, os dados foram registrados no questionário do paciente.

As amostras de sangue foram coletadas por um profissional qualificado na sala de coleta da unidade de diálise, utilizando o acesso para hemodiálise. Após a coleta, o sangue foi centrifugado e as amostras de plasma e eritrócitos foram congeladas a −20ºC para análise do perfil oxidativo.

Trezentos microlitros de plasma foram adicionados a 600 μL de etanol absoluto resfriado (4 °C) para determinação dos níveis de nitrito no plasma. A mistura foi agitada vigorosamente, de modo que o etanol gelado precipitasse as proteínas presentes no plasma. Após a agitação, o plasma com etanol foi mantido refrigerado a 0 °C por 30 minutos e, em seguida, as amostras foram centrifugadas a 10.000 rpm por 5 minutos. Posteriormente, o sobrenadante foi aliquotado e congelado para análise posterior. A dosagem de óxido nítrico foi realizada utilizando o método de Miranda^9^, seguindo o ensaio de Griess. Este método requer a preparação de dois reagentes (Reagente A: 0,5 g de dicloridrato de naftil etilenodiamina em 500 mL de água destilada e Reagente B: 5 g de sulfanilamida, 25 mL de ácido fosfórico), que foram misturados somente no dia do ensaio. Uma solução padrão foi preparada por meio da adição de 2 mg de nitrito de sódio em 50 mL de água destilada (1,38 mL da solução estoque + 0,62 mL de água). Em seguida, 100 μL da amostra e da solução de trabalho foram pipetados nos poços correspondentes, em uma placa de 96 poços, aos quais foram adicionados 100 μL do reagente de Griess. A mistura foi incubada por 15 minutos e, posteriormente, a leitura foi realizada em espectrofotômetro a 550 nm.

Os níveis de substâncias reativas ao ácido tiobarbitúrico (TBARS) foram determinados de acordo com o método descrito por Ohkawa et al.^10^. Alíquotas de soro e lise celular (0,2 mL) foram adicionadas a 250 µL de ácido acético (2,5M; pH 3,4) e 250 µL de ácido tiobarbitúrico (0,8%). Esta mistura foi incubada durante 90 minutos a 95 °C e, em seguida, centrifugada a 5.000 g por 15 minutos. O sobrenadante da reação foi coletado e analisado em um leitor de microplacas a 532 nm.

O status antioxidante não enzimático do plasma foi avaliado através da redução do brometo de 3-[4,5-dimetiltiazol-2-il]-2,5-difeniltetrazólio (MTT) por moléculas com ação antioxidante, de acordo com Mosmann^11^. Em um tubo de ensaio, foram adicionados 100 µL de amostra e 20 µL de MTT com 380 µL de solução salina tamponada com fosfato (10 mmol/L; pH 7,4). Esta solução foi incubada em banho-maria por 60 minutos a 37°C, protegida da luz. Em seguida, adicionou-se 1 mL de uma solução de álcool isopropílico com ácido clorídrico (0,4 N) que foi centrifugada a 3500 rpm por 10 minutos à temperatura ambiente. O sobrenadante da reação foi transferido para uma placa de 96 poços e analisado no leitor de microplacas (Spectra Max 250; *Molecular Devices*) a 570 nm. A avaliação do status antioxidante foi expressa de acordo com a absorbância obtida.

O conteúdo de espécies reativas de oxigênio (ROS) foi avaliado no plasma por meio da intensidade de fluorescência do corante sensível ao redox 2′,7′‐diclorodihidrofluoresceína diacetato (DCFH, 100 µM; *Sigma-Aldrich*). O DCFH é capaz de atravessar a membrana celular e sofre um processo de desacetilação por enzimas esterase intracelulares, formando o composto intermediário 2′,7′-diclorodihidrofluoresceína no citoplasma, que reage com o ROS formando 2′,7′-diclorofluoresceína oxidada (DCF), uma molécula fluorescente. O ensaio envolveu a adição de 30 µL de amostra, 30 µL de solução salina tamponada com fosfato (10 mmol/L; pH 7,4) e 24 µL de DCF (10 µM) em uma placa de 96 poços. A placa foi incubada por 30 minutos a 37 °C. A fluorescência foi medida utilizando um leitor de microplacas (Spectra Max 250; *Molecular Devices*; EUA) com comprimentos de onda de excitação e emissão definidos em 485 e 535 nm, respetivamente. A quantidade de ROS intracelular foi expressa como intensidade de fluorescência.

### Análise Estatística

A normalidade foi verificada utilizando o teste de Kolmogorov-Smirnov. As variáveis qualitativas foram descritas por frequência absoluta e relativa, e as variáveis quantitativas por média ± desvio padrão e mediana.

O teste de Wilcoxon foi utilizado para comparar os marcadores pré e pós-hemodiálise, e o teste de McNemmar foi empregado para comparar os marcadores pré e pós-AF. Para estabelecer o ponto de corte da albumina capaz de predizer o óbito, utilizou-se a curva ROC. Para a análise de sobrevida dos pacientes, o tempo de acompanhamento foi calculado em meses, desde a data da BIA/avaliação até a data do óbito/transplante/encerramento. O desfecho de interesse foi o óbito, sendo considerados censurados os pacientes transplantados e vivos. Os pacientes que abandonaram o estudo, foram transferidos ou estavam ausentes por outros motivos não foram incluídos nesta análise, uma vez que não havia registro de dados. As curvas de Kaplan-Meier foram geradas utilizando o teste log-rank para comparar a sobrevida de acordo com os fatores de interesse. As estimativas do *hazard ratio* com IC 95% foram obtidas por meio da análise de regressão de Cox. Para a análise multivariada, foram selecionadas - a partir da análise univariada - as variáveis com valores de p < 0,10 e que tiveram poucos dados perdidos. Modelos adicionais de regressão de Cox foram desenvolvidos utilizando os valores finais ou os últimos valores registrados de albumina e glicose, com o objetivo de explorar preditores bioquímicos de mortalidade em estágio avançado. Os pacientes foram categorizados com base em pontos de corte clínicos padrão: albumina < 3,9 g/dL e glicose ≥ 126 mg/dL. Além disso, o delta (pós-pré) da albumina e do AF foram comparados entre sobreviventes e não sobreviventes usando análise descritiva e gráfica, incluindo gráficos de violino e gráficos de barras agrupadas. As análises estatísticas foram realizadas no programa estatístico SPSS 25.0 (SPSS Inc., EUA). O nível de significância adotado foi de 0,05.

## Resultados

O presente estudo analisou 82 pacientes com DRC em hemodiálise (51,2% do sexo masculino). Após 36 meses de acompanhamento, 15 interromperam o tratamento em decorrência de transplante renal, 7 foram transferidos e 35 foram à óbito. As comorbidades associadas foram hipertensão (91,5%), insuficiência cardíaca (17,1%) e obesidade (24,4%). O tempo médio de sobrevida foi de 20,3 meses em pacientes com diabetes e de 26,4 meses em pacientes sem diabetes; os pacientes diabéticos apresentaram uma taxa de mortalidade 22,6% maior do que o grupo sem diabetes. Entre os 42 pacientes com valores de glicose disponíveis ao final do acompanhamento, 11 tinham diagnóstico prévio de diabetes mellitus. Esses indivíduos apresentaram glicemia final média de 151,4 ± 49,9 mg/dL, enquanto os não diabéticos (n = 31) apresentaram média de 95,9 ± 46,2 mg/dL. Esses achados corroboram a consistência entre o diagnóstico clínico e os marcadores bioquímicos do estado glicêmico.

A taxa de sobrevida dos pacientes não diabéticos e diabéticos foi de 94,7% e 78,6% aos seis meses, 81% e 64,3% aos 12 meses, 58,3% e 35,7% aos 24 meses e 49,7% e 28,6% aos 36 meses. A Tabela 1 descreve a caracterização geral da amostra coletada no tempo zero, ou seja, pré-diálise, no início do acompanhamento.

**Tabela 1:** Características basais dos pacientes com DRC

Variáveis	n	Média ± DP	Mediana (mínimo–máximo)
Idade (anos)	82	52,5 ± 17,4	54,5 (20–84)
Tempo em diálise (meses)	82	54,2 ± 51,6	40,5 (3–202)
Peso pós-diálise (kg)	82	68,4 ± 19,7	63,1 (36,8–119,8)
IMC pós-diálise (Kg/m^2^)	82	25,9 ± 5,9	25 (16,6–41,9)
Albumina (g/dL)	82	3,8 ± 0,3	3,8 (2,9–4,6)
Proteína C Reativa (mg/dL)	65	12,2 ± 15,3	7,8 (0,23–84)
PCR/albumina	65	3,2 ± 4,0	1,8 (0,1–22,1)
Triglicerídeos (mg/dL)	79	148,2 ± 88,5	125 (15–515)
HDL (md/dL)	79	42,1 ± 15,6	39 (11–88)
TRIG/HDL	79	4,2 ± 3,4	3,3 (0,4–21,7)

Abreviaturas – DP: Desvio Padrão; kg: quilograma; IMC: Índice de Massa Corporal; PCR: Proteína C Reativa; HDL: Lipoproteína de Alta Densidade.

A Tabela 2 apresenta as variações encontradas nos marcadores biológicos analisados antes e após a sessão de hemodiálise no tempo zero. O marcador tiol apresentou um aumento significativo (p = 0,001), o marcador MTT apresentou uma redução significativa (p = 0,031) e o marcador TBARS também apresentou uma diminuição significativa (p < 0,001). Em contraste, o marcador DCF apresentou um aumento significativo (p = 0,046). O marcador óxido nítrico não apresentou diferença significativa. A Tabela 2 também demonstra um aumento significativo no AF em pacientes após a hemodiálise.

**Tabela 2:** Comparação entre marcadores de dano oxidativo e ângulo de fase antes e após a sessão de hemodiálise no início do estudo

Marcador	N	Antes	Após	p
		Mediana (Min–Max)	Mediana (Min–Max)	
Tióis (nmol)	73	14,9 (3,3; 45,8)	17,8 (4,9; 61,1)	**0,001**
MTT (O.D.)	75	0,39 (0,15; 1,92)	0,32 (0,12; 2,18)	**0,031**
TBARS (nmol/mL)	73	552,4 (12,4; 1377,7)	214,1 (15,3; 1108,2)	**<0,001**
DCF (intensidade de fluorescência)	73	295,8 (195; 568,2)	321,8 (116,8; 888,3)	**0,046**
NO (mg/L)	39	40,3 (29,3; 65)	39,7 (27,9; 73,1)	0,132
Ângulo de fase	82	5,2 (2,4; 7,7)	5,9 (2,7; 10,6)	**<0,001**

Abreviaturas – DCF: diclorodihidrofluoresceína; TBARS: substâncias reativas ao ácido tiobarbitúrico; MTT: (3-(4,5-dimetiltiazol-2-il)-2,5-difeniltetrazólio brometo); NO: óxido nítrico. Notas – p: teste de Wilcoxon; nível de significância = 0,05.

Além disso, 11,0% dos pacientes apresentaram AF alterado (<4) no início do acompanhamento. Antes da hemodiálise, 14 pacientes (17,1%) apresentaram AF < 4 e, após a sessão, 9 (11%) permaneceram com AF < 4 (p = 0,063). No entanto, mais de 1/3 (5/14) dos pacientes que inicialmente apresentaram AF < 4 obtiveram melhora. O tempo médio de sobrevida dos pacientes com AF < 4 foi de 11,6 meses (IC 95%: 6,5 – 16,8), e o dos pacientes com AF ≥ 4 foi de 27,0 meses (IC 95%: 24,2 – 29,8), p < 0,001, com diferença significativa (log-rank = 11,5; p < 0,001; HR = 5,66 (IC 95%: 2,44 – 13,12). As taxas de sobrevida em AF < 4 e AF ≥ 4 foram de 66,75% × 95,1%, 55,6% × 80,9%, 11,1% × 60,2% e 11,1% × 50,6% aos 6, 12, 24 e 36 meses, respectivamente. A Tabela 3 apresenta as variáveis que contribuíram significativamente para o HR de óbito ao longo dos 36 meses.

**Tabela 3:** Biomarcadores que contribuem significativamente para o *hazard ratio*, considerando o ângulo de fase antes e após a hemodiálise

		Univariada				Multivariada			
		p	HR	IC95%		p	HR	IC95%	
ângulo de fase antes da hemodiálise	Idade (cada 5 anos)	0,003	1,19	1,06	1,35	0,172	1,10	0,96	1,27
	Diabetes	0,091	1,88	0,90	3,93	0,692	0,84	0,34	2,03
	Pós AF < 4	**0,000**	**5,99**	**2,80**	**12,79**	**0,042**	**2,69**	**1,03**	**7,01**
	DCF pós (cada 50 uM)	0,076	0,87	0,75	1,01	0,220	0,90	0,76	1,07
	Albumina < 3,9	**0,001**	**4,39**	**1,81**	**10,63**	**0,017**	**3,40**	**1,24**	**9,30**
ângulo de fase após a hemodiálise	Idade (cada 5 anos)	0,003	1,19	1,06	1,35	0,122	1,12	0,97	1,31
	Diabetes	0,091	1,88	0,90	3,93	0,638	0,81	0,33	1,96
	Pré AF < 4	**0,000**	**5,66**	**2,44**	**13,12**	0,285	1,92	0,58	6,37
	DCF pós (cada 50 uM)	0,076	0,87	0,75	1,01	0,174	0,89	0,75	1,05
	Albumina < 3,9	**0,001**	**4,39**	**1,81**	**10,63**	**0,008**	**3,81**	**1,41**	**10,25**

Abreviaturas – DCF: diclorodihidrofluoresceína; AF: ângulo de fase; HR: razão de risco. Notas – pré: antes da hemodiálise; pós: após a hemodiálise; Albumina < 3,9: ponto de corte; p: análise univariada ou multivariada; IC95%: intervalo de confiança 95%; Nível de significância = 0,05.

O tempo médio de sobrevida em pacientes com albumina < 3,9 foi de 21,2 meses (IC 95%: 17,8–24,7), e o de pacientes com albumina ≥ 3,9 foi de 31,7 meses (IC 95%: 28,2–35,2), com diferença significativa (log-rank = 13,0; p < 0,001; HR = 4,39; IC 95%: 1,81 – 10,63). Além disso, 57,3% apresentaram albumina < 3,9 (AUC = 0,742, IC 95%: 0,635– 0,849; p < 0,001; sensibilidade = 82,7%; especificidade = 61,7%). Quanto à sobrevida, as taxas aos 6, 12, 24 e 36 meses para pacientes com albumina ≥ 3,9 e < 3,9 foram de 96,6% e 88,2%, 88,5% e 70,7%, 84,1% e 35,4%, e 75,0% e 27,6%, respectivamente.

Em uma análise secundária, utilizando valores medidos mais próximos ao óbito ou ao final do acompanhamento, os pacientes com níveis de albumina < 3,9 g/dL apresentaram um risco significativamente aumentado de mortalidade (HR = 2,66; p < 0,001). Esses achados reforçam o valor prognóstico da albumina no início do estudo e nos estágios finais do acompanhamento e confirmam a classificação do diabetes utilizando valores glicêmicos terminais. Embora a albumina tenha apresentado pequenas flutuações ao longo do acompanhamento, seus valores finais foram semelhantes aos basais (média: 3,78 ± 0,73 g/dL; mediana: 3,90; variação: 1,3–5,4 g/dL). Embora a glicose não tenha sido incluída nos modelos de sobrevida, seus valores finais corroboraram o diagnóstico de diabetes mellitus nos pacientes classificados como diabéticos.

Não houve diferença significativa nas curvas de sobrevida de acordo com a presença de DM (log-rank = 3,0; p = 0,083; HR = 1,88; IC 95%: 0,90–3,93). O tempo médio de sobrevida em pacientes com DM foi de 20,3 meses (IC 95%: 14,1–26,6), e o de pacientes sem DM foi de 26,4 meses (IC 95%: 23,4–29,5). Esses achados destacam a relevância prognóstica da hiperglicemia terminal, mesmo em pacientes não previamente classificados como diabéticos. A Figura 2 mostra a sobrevida global ao longo de 36 meses.

**Figura 2:** Curvas de sobrevida de Kaplan-Meier aos 36 meses para pacientes com doença renal crônica em hemodiálise. (A) Taxa de sobrevida global da coorte; (B) Comparação da sobrevida entre pacientes diabéticos (DM) e não diabéticos; (C) Sobrevida estratificada pelo ponto de corte do ângulo de fase (AF), evidenciando diferenças significativas entre pacientes com AF < 4 e AF ≥ 4. A significância estatística foi definida como p < 0,05 para todas as comparações.

Entre os 35 pacientes que foram a óbito, 30 apresentaram causa conhecida, que foi categorizada segundo a etiologia: colapso cardiovascular/circulatório (n = 11), infecciosa/inflamatória (n = 10), neoplásica (n = 3), renal/falha de acesso (n = 2), gastrointestinal/hemorrágica (n = 1), fim da vida/paliativa (n = 1) e não classificada (n = 3). O tempo médio até o óbito variou de acordo com a causa, apresentando sobrevida mais curta em óbitos por causas infecciosas e cardiovasculares (16,5 e 17,7 meses, respectivamente) e mais longa em eventos neoplásicos ou hemorrágicos.

## Discussâo

Durante o acompanhamento dos pacientes, a taxa de sobrevida foi de 91,4% aos 6 meses, 77,6% aos 12 meses, 53,5% aos 24 meses e 45,2% aos 36 meses. Essas taxas de sobrevida foram semelhantes às descritas pelo Censo Brasileiro de Diálise, onde a taxa de sobrevida descrita para o ano de 2021 foi de 77,7%^12^.

Foi observada uma diferença significativa nos parâmetros de tióis, TBARS e MTT ao comparar as medições pré-diálise com as pós-diálise, conforme apresentado na Tabela 2. A homeostase prejudicada dos tióis sanguíneos tem sido comumente descrita na doença renal crônica, com o nível mais elevado de expressão nos pacientes em estágio terminal submetidos à hemodiálise. Os fatores envolvidos nos níveis anormais de tiol podem incluir metabolismo prejudicado do tiol nos rins e outros tecidos expostos a toxinas urêmicas, bem como consequências metabólicas da comorbidade urêmica, incluindo inflamação crônica, desnutrição, anemia e estresse oxidativo^13^.

A determinação de TBARS é amplamente utilizada para monitorar a peroxidação lipídica. Nosso estudo observou uma redução significativa nos níveis de TBARS ao comparar os momentos pré e pós-diálise. A hemodiálise apresentou um efeito significativo na redução do estresse oxidativo em nossa pesquisa, diferentemente do estudo realizado por Zargari and Sedighi^14^, no qual as concentrações mais elevadas de TBARS foram observadas após a HD. Outro estudo, realizado por Schettler et al., também observou um aumento nos níveis de TBARS após a hemodiálise. No entanto, quando as concentrações de TBARS foram corrigidas quanto ao colesterol plasmático, a fim de considerar a hemoconcentração, observou-se uma redução significativa do TBARS devido ao tratamento de diálise, tanto no plasma venoso quanto no arterial^15,16^. Nossos achados revelaram um aumento nos níveis de DCF após a hemodiálise, o que pode ser atribuído à eriptose induzida por uremia, um fenômeno caracterizado pela redução do tamanho dos eritrócitos, formação de bolhas na membrana e transferência de fosfatidilserina para a camada externa da membrana^17,18^. Após a sessão de hemodiálise, o ensaio MTT indicou uma redução significativa dos níveis de oxidantes, o que pode estar relacionado com uma diminuição dos níveis de oxidação, uma vez que o MTT é capaz de detectar a geração de superóxido^19^.

A taxa de sobrevida dos pacientes com AF inferior a 4° - um marcador de desnutrição para todos os pacientes, independentemente do sexo - também foi avaliada. A sobrevida nesse grupo foi de 66,7% aos 6 meses, 55,6% aos 12 meses e 11,1% aos 24 meses. No grupo que não apresentou desnutrição, a sobrevida foi de 95,1%, 80,9% e 60,2%, respectivamente. O parâmetro que apresentou maior associação com a mortalidade foi a desnutrição, medida pelo AF (p < 0,001). Em estudo realizado por Ruperto e Barril^20^, observou-se que um AF inferior a 4° atuou como preditor de mortalidade, com mais de 70% dos não sobreviventes apresentando AF < 4°^21,22^. Zeni et al.^21^, em estudo realizado no mesmo centro e com os mesmos pacientes, observaram que o AF permaneceu associado ao óbito após ajuste para idade, razão água extracelular/água corporal total, raça, insuficiência cardíaca, obesidade, diabetes, hipertensão e colesterol HDL^21^.

Como o AF avalia a função elétrica das membranas celulares, um AF baixo está associado à morte celular ou à redução da função celular, sendo também um sinal de sarcopenia e desnutrição, o que pode afetar significativamente a qualidade de vida e a sobrevida de pacientes em diálise^23–26^. Esses achados sugerem que o AF pode ser um dos melhores preditores de mortalidade em pacientes em hemodiálise. Se essa deficiência fosse corrigida, a mortalidade poderia ser reduzida em 50% ao longo de 24 meses.

A taxa de sobrevida dos pacientes com diabetes no USRDS em 2011 foi de 92,1% em três meses, 78,3% em 12 meses e 66,8% em 24 meses^27^. No trabalho de Silva et al., observou-se que a sobrevida de indivíduos não diabéticos foi diferente em relação aos diabéticos: 92% vs. 87% em 1 ano, 87% vs. 77% em 2 anos e 69% vs. 50% em 5 anos^28^. Zeng et al.^26^ realizaram um estudo com 87 pacientes em hemodiálise, dos quais 33 eram diabéticos e 54 não diabéticos^26^.

A albumina foi a única variável independente que influenciou significativamente a mortalidade dos pacientes. Baixos níveis séricos de albumina na diálise peritoneal reduzem a sobrevida em pacientes com doença renal em estágio V^29^, indicando que a variação elevada dos níveis séricos de albumina está associada ao aumento da mortalidade por todas as causas^30^. No presente estudo, o risco de óbito durante o acompanhamento foi 4,4 e 3,8 vezes maior quando a albumina foi inferior a 3,9 g/dL nas análises univariada e multivariada, respectivamente. No estudo de Fulks et al.^31^, níveis de albumina > 4,8 g/dL estiveram associados a reduções de 15 a 18% no risco relativo de mortalidade em uma população não selecionada de homens na faixa etária de 20 a 49 anos. O estudo DOPPS (*Dialysis Outcomes and Practice Patterns Study*)^32–34^ também sugere que >60% dos pacientes em HD apresentam níveis de albumina < 4,0 g/dL. Baixas concentrações séricas de albumina têm se mostrado consistentemente um poderoso preditor de morbimortalidade em populações com DRC. De modo geral, manter níveis séricos adequados de albumina e minimizar as flutuações pode desempenhar um papel crucial na melhoria dos desfechos de sobrevida de pacientes em hemodiálise.

Também exploramos o impacto de marcadores bioquímicos em estágio avançado na mortalidade. Níveis reduzidos de albumina e hiperglicemia próximos ao óbito mantiveram-se fortemente associados à redução da sobrevida, reforçando a importância do monitoramento metabólico de rotina ao longo do acompanhamento.

Além disso, observamos que pacientes com melhores respostas agudas à sessão inicial de hemodiálise apresentaram maior probabilidade de sobrevida, conforme mensurado pelo aumento do AF e por elevações leves nos níveis de albumina. Esses achados sugerem que o controle metabólico a longo prazo e a resiliência fisiológica imediata desempenham um papel importante nos desfechos de sobrevida em pacientes em HD.

Em relação à causa do óbito, as etiologias mais comuns foram cardiovasculares e infecciosas, em consonância com a literatura existente e associadas à mortalidade precoce. A ausência de registros estruturados nos prontuários médicos limitou uma estratificação mais detalhada, evidenciando a necessidade de uma melhor documentação clínica em estudos futuros.

## Data Availability

Os conjuntos de dados gerados e analisados durante o presente estudo não estão disponíveis publicamente devido a restrições éticas e de privacidade relacionadas aos dados dos pacientes, mas podem ser obtidos com o autor correspondente mediante solicitação razoável e com as devidas autorizações institucionais.
